# Synthesis, Characterization, Theoretical Crystal Structure, and Antibacterial Activities of Some Transition Metal Complexes of the Thiosemicarbazone (Z)-2-(pyrrolidin-2-ylidene)hydrazinecarbothioamide

**DOI:** 10.1155/2011/483101

**Published:** 2011-07-24

**Authors:** Ahmed A. Al-Amiery, Yasmien K. Al-Majedy, Haziem Abdulreazak, Hussain Abood

**Affiliations:** ^1^Biotechnology Division, Applied Science Department, University of Technology, Baghdad 10066, Iraq; ^2^Department of Chemical and Processing Engineering, Faculty of Engineering and Built Environment, University of Kebangsaan Malaysia, Bangi, Selangor 43600, Malaysia; ^3^Chemistry Department, College of Science, Al Koofa University, Al Koofa 54003, Iraq

## Abstract

*Problem Statement*. In Iraq like most third world countries, attempts discovered new antibiotic drugs derived from thiosemicarbazide and its metal complexes and developed the branch of applied in organic chemistry. *Approach*. New (Z)-2-(pyrrolidin-2-ylidene)hydrazinecarbothioamide (L) was synthesized in a good yield by the reaction of pyrrolidone with thiosemicarbazide. Co(II), Ni(II), and Cu(II) complexes of (L) were prepared and characterized by FT-IR, UV/visible spectra, ^1^HNMR, and CHN analyses. Moreover, charge, bond length, bond angle, twist angle, heat of formation, and steric energy were calculated by using of the ChemOffice program, and the DFT calculations for the complexes were done. The free ligand and its metal complexes were tested *in vitro* against several microorganisms (*Staphylococcus aurous*, *E. coli*, *Proteus vulgaris*, *Pseudomonas*, and *Klebsiella pneumoniae*) to assess their antimicrobial properties. *Results*. The study shows that these complexes have octahedral geometry; in addition, it has high activity against tested bacteria. *Conclusion/Recommendations*. Based on the reported results, it may be concluded that ligand acts as bidentate, neutral ligand, coordinating through one of the nitrogen and sulfur atoms.

## 1. Introduction

The chemistry of thiosemicarbazones has received considerable attention because of their variable bonding modes, promising biological implications, structural diversity, and ion-sensing ability [1–3]. They have been used as drugs and are reported to possess a wide variety of biological activities against bacteria, fungi, and certain type of tumors, and they are also a useful model for bioinorganic processes [4–6]. The activity of these compounds is strongly dependent on the nature of the heteroatomic ring and the position of attachment to the ring as well as the form of thiosemicarbazone moiety [7]. These are studied extensively due to their flexibility, selectivity, and sensitivity towards the central metal atom, and structural similarities with natural biological substances, and due to the presence of imine group (−N=CH−) which imparts the biological activity [8–12]. There is no report on the complexes of (Z)-2-(pyrrolidin-2-ylidene)hydrazinecarbothioamide (L). We now present details of the complexes of (L) with cobalt(II), nickel(II), and copper(II) ion.

## 2. Materials and Methods

### 2.1. General

All chemicals used were of reagent grade (supplied by either Merck or Fluka) and used as supplied. The FTIR spectra in the range (4000–200) cm^−1^ were recorded as cesium iodide disc on FTIR 8300 Shimadzu Spectrophotometer. Proton NMR spectra were recorded on Bruker-DPX 300 MHz spectrometer with TMS as internal standard. The UV-Visible spectra were measured in ethanol using Shimadzu UV-Vis. 160 A spectrophotometer in the range (200–1000) nm. Magnetic susceptibility measurement for complexes was obtained at room temperature using (magnetic susceptibility balance model MSB-MKI). Flame atomic absorption of elemental analyzer, shimadzu AA-670, was used for metal determination. Elemental microanalysis, was carried out using C.H.N elemental analyzer model 5500-Carlo Erba instrument. Gallen Kamp M.F.B.600.010 F melting point apparatus was used to measure the melting point of all the prepared compounds.

### 2.2. Synthesis of (Z)-2-(pyrrolidin-2-ylidene)hydrazinecarbothioamide (L)

**Scheme 1:**
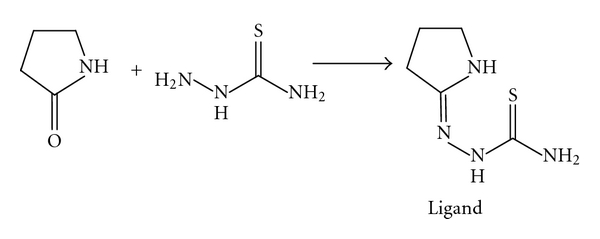


Pyrrolidone (10 mmol) in hot ethanol (20 mL) was mixed with hot ethanolic solution of thiosemicarbazide (10 mmol). The mixture was refluxed for 6 hours on a water bath; on cooling the contents, the precipitate was separated out, filtered washed with 50% cold ethanol, and dried in vacuum over P_4_O_10_, melting point of 176°C and yield of 73% of yellow crystal (Z)-2-(pyrrolidin-2-ylidene)hydrazinecarbothioamide. The proposed structure can be shown according to Scheme 1.

Proton NMR (1.4 t(2H) for CH_2_, m. 2.0(2H) for CH_2_, t. 2.9(2H) for CH_2_, 2.4 for NH, 8.7 for NH_2_, 10.8 for NH). Elemental analysis found % C 37.10(37.95), H 6.12(6.37), N 34.85(35.41) calculated for C_5_H_10_N_4_S.

### 2.3. Synthesis of the Complexes

Metal salts (5 mmol) in hot ethanol (20 mL) were mixed with hot ethanolic solution of the ligand (10 mmol) and refluxed for 4 hours on a water bath; on cooling the contents, the colored complexes separated out in each case. The product was filtered, washed with cold 50% ethanol, and dried in vacuum over P_4_O_10_. Purity of the complexes was checked by thin layer chromatography (TLC).

### 2.4. Study of Complex Formation in Solution

The complexes of the ligand with metal ions were studied in DMF, in order to determine the M : L ratio in the complex following the molar ratio method. A series of solutions were prepared having a constant concentration (10^−3^ M) of the metal ion and (L). The M : L ratio was determined from the relationship between the absorption of light and the molar ratio of M : L. The results of complexes formation in solution were listed in Table 1.

### 2.5. The Biological Activity for the Ligand and Its Complexes

All tests with the microorganisms were obtained from the Biotechnology Division, Department of Applied Science, University of Technology, which were as follows: *Escherichia coli*, *Klebsiella pneumoniae*, *Proteus vulgaris*, *Pseudomonas aeruginosa*, and *Staphylococcus aureus*. Antibacterial activities of the ligand and the soluble complexes were evaluated by the disc diffusion technique [13]. Filter paper (Whatman no. 4) discs (6 mm diameter) were soaked in a solution of the test compounds of 10^−2^ × 5 mg mL^−1^ concentration in DMF and placed on nutrient agar plates after drying to remove the solvent. The inhibition zones were measured after 24 h. DMF was used as control. The results are shown in Figure 2.

## 3. Results

**General**

The complexes were synthesized by reacting ligand with the metal ions in 1 : 2 molar ratio in ethanolic medium.

### 3.1. Infrared Spectroscopy

**Figure 1:**
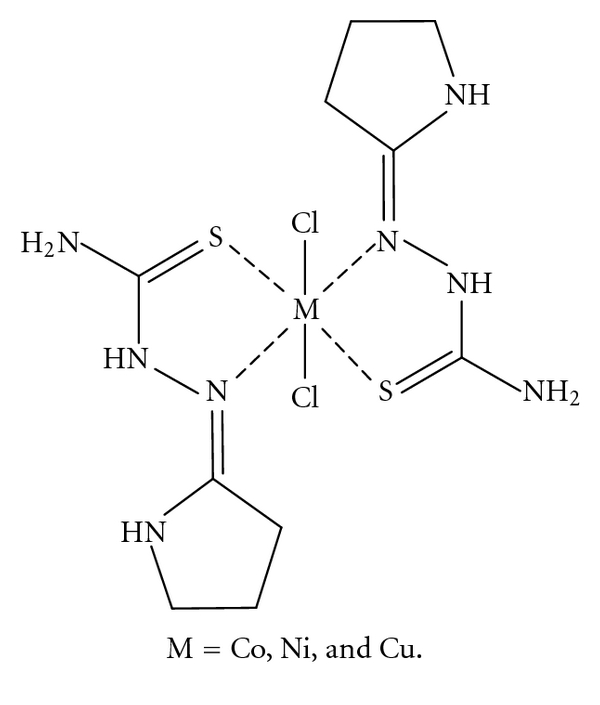
Structures of the complexes.

A study and comparison of infrared spectra of free ligand and its metal complexes (Table 2) infers that the ligand behaves as neutral bidentate and its metals are coordinated through N and S of the thio-keto group (Figure 1).

Strong bands in the 3396 and 3275 cm^−1^ regions were observed and attributed to N–H vibrations in the ligand. The negligible effect on these frequencies after complexation precludes the possibility of complexation at this group. The absorption at 1600 cm^−1^ in free ligand can be attributed to (C=N) stretching vibration of imine nitrogen, which is in a good agreement with previous observations. On complexation, this frequency was observed to be shifted to a lower wave number (Table 2). These observations suggest the involvement of unsaturated nitrogen atoms of the azomethine groups in bonding with the metal ions. Coordination of sulfur with the metal ion would result in the displacement of electrons towards the latter, thus resulting in the weakening of the (C=S) bond. Hence, on complexation (C=S) stretching vibrations should decrease and those of (C–N) should increase. The IR spectral bands in the ligand are practically unchanged in the complexes but show some new bands with medium to weak intensity in the 520–440 cm^−1^ region tentatively assigned to (M–N) and (M–S). (M–Cl) is tentatively assigned in the 360–350 cm^−1^ region [14, 15].

### 3.2. Magnetic Susceptibility

At room temperature, all the complexes under study show the magnetic moment in the range of 2.1–4.7 B.M. corresponding with the unpaired electrons. The magnetic moment of Co(II) complex is 4.1 BM showing that the Co(II) complex is typically a high spin complex and having octahedral structure [16]. For Ni(II) complex, the observed magnetic moment value is 3.0 BM which is well within the expected range for Ni(II) complex with octahedral stereochemistry (2.83–4.0 BM). The magnetic moment for Cu(II) complex is 2.0 BM. The reported values for the Cu(II) complex have no major spin orbital interactions (1.75–2.20 BM) [16, 17]. Thus the present Cu(II) complex is without any spin orbital interaction with octahedral geometry. In octahedral Cu(II) complex, the ground state is 2Eg, and large spin orbital contribution to the magnetic moment is expected.

## 4. UV-Visible Spectroscopy

### 4.1. Cobalt(II) Complexes

The electronic spectra of Co(II) complex recorded in DMF solution display three absorption bands at 8100, 16500, and 22000 cm^−1^ corresponding to the following transitions, respectively: ^4^T_1g(F)_→^4^T_2g(F)_, ^4^T_1g(F)_→^4^A_2g(F)_, and ^4^T_1g(F)_→^4^T_1g(P)_. This shows that these complexes have octahedral geometry.

### 4.2. Nickel(II) Complexes

The electronic spectra of the Ni(II) complex show absorption bands at 9000, 13000, and 24000 cm^−1^ and may be assigned to the transitions ^3^A_2_g→^3^T_2_g, ^3^A_2_g→^3^T_1g(F)_, and ^3^A_2g(F)_→^3^T_1g(P)_ corresponding with an octahedral geometry.

### 4.3. Copper(II) Complex

The Cu(II) complex under study displays absorption bands 13400 and 16000 cm^−1^. These bands were assigned to the following transitions from a distorted octahedral geometry [18]: ^2^B_1_g → ^2^A_1_g, ^2^B_1_g → ^2^B_2_g.

### 4.4. Stereo Suggested Structures of Complexes

According to the above-mentioned data (spectra, molar conductance, molar ratio, and magnetic properties), the proposed structures of completes were shown as in Figure 1.

## 5. Biological Activity

It is known that chelation tends to make the ligand act as more powerful and potent bactericidal agents, thus killing more of the bacteria than the ligand. It is observed that, in a complex, the positive charge of the metal is partially shared with the donor atoms present in the ligands, and there may be *π*-electron delocalization over the whole chelate [19]. This increases the lipophilic character of the metal chelate and favours its permeation through the lipoid layer of the bacterial membranes. The increased lipophilic character of these complexes seems to be the reason of their enhanced potent antibacterial activity. There are other factors which also increase the activity, which are solubility, conductivity, and bond length between the metal and the ligand.

## 6. Stability Study

These data show that the atomic charge was affected by the presence of substituent of rings as shown in Tables 3, 4, and 5. As a reference compound, the unsubstituted ligand (Figure 3), the data for minimized geometry, and the 3d-geometrical structure are shown in (Figure 3). 

## 7. Discussion

**Figure 2:**
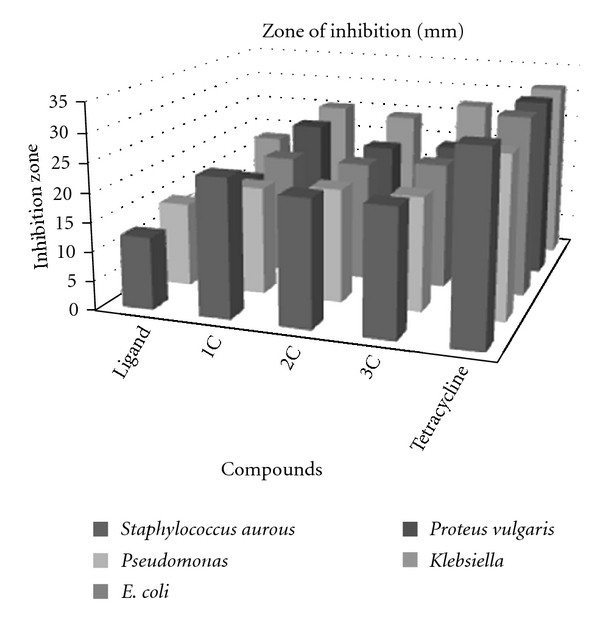
Antibacterial activity of the ligand and its metal complexes.

**Figure 3:**
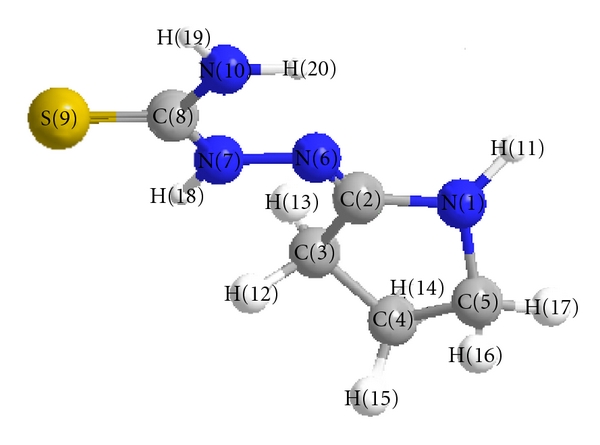
The 3d-geometrical structure for the ligand.

All the solid complexes are stable in air. The metal complexes are soluble in DMF and DMSO. Table 1 shows the colors, elemental analyses, and molar conductivities of the cobalt(II), nickel(II), and copper(II) complexes. Thiosemicarbazones can coordinate with metal ions as neutral ligand. The C, H, and N data indicate that the complexes of neutral ligand are coordinated to the metal ion, and the other coordination positions were occupied by chlorine, as confirmed by the IR spectra of the complexes. The antibacterial screening (Figure 2) indicates that the complexes inhibit the growth of *Staphylococcus aurous* and *Klebsiella*, whereas other complexes have less activity against other tested bacteria. The theoretical data obtained show that the heat of formation is 52.61737 kcal/mole, and the highest atomic charge (Table 5) in ligand molecule is at [S4 −0.696] and the next charge value is at [N1 −0.431]. These data show clearly that these atoms are the most reactive toward the bonding with the metal. The determined bond angle (Table 4) and 3D-geometrical (Figure 3) structure indicate that this molecule is planar. The stability for the prepared complexes was studied theoretically by the density function theory (DFT). The total energy for the complexes was calculated, and it was shown that the copper complex is the most stable, and the cobalt complex is the least stable as follow: Cu-complex > Ni-complex > Co-complex.

## 8. Conclusion

Based on the reported results, it may be concluded that ligand acts as bidentate neutral ligand, coordinating through one of the nitrogen atoms and the sulfur. In the present work, which facilitates the formation of six member rings, this shifts the nitrogen atom of the other ring away from the coordination site. In the present investigations, all the complexes are found to be mononuclear, based on the FT-IR spectral data. The coordination number six is attained by coordination with the two bidentate ligand molecules and two chloride atoms. Based on the physicochemical and the spectral studies the tentative structures proposed for the complexes are shown in Figure 1.

## Figures and Tables

**Table 1:** Conductivity, colors, and elemental analysis data of the complexes.

NO.	Complex	Yield %	M.P. °C	Analysis: Found (cal.) %				M : L	Color	Ωm ohm^−1^ cm^2^ mol^−1^
				Metal	C	H	N			
C1	Co(C_5_H_10_N_4_S)_2_Cl_2_	75	Over 300	13.21 (12.7)	24.91 (26.4)	4.52 (4.22)	24.11 (24.87)	1 : 2	Dark brown	30
C2	Ni(C_5_H_10_N_4_S)_2_Cl_2_	60	Over 300	13.16 (12.65)	23.93 (25.12)	4.52 (4.12)	25.12 (24.16)	1 : 2	Light green	65
C3	Cu(C_5_H_10_N_4_S)_2_Cl_2_	77	260	14.09 (13.39)	23.64 (25.94)	4.47 (4.30)	23.85 (24.43)	1 : 2	Green	30

**Table 2:** Infrared absorption frequencies (cm^−1^) of Co(II), Ni(II), and Cu(II) complexes.

Compounds	*ν*(N–H)	*ν*(C=N)	*ν*(C–N)	*ν*(N–N)	*ν*(C=S)	*ν*(M–N)	v(M–S)	v(M–Cl)
Ligand	3395 s, 3275 s	1600 s	1330 s, 1200 m	1035 m	855 s, 770 s	—	—	—
C1	3330 m, 3265 m	1570 s	1380 s, 1240 m	1075 m	850 s, 780 m	525 m	440 m	350 w
C2	3320 m, 3260 m	1565 s	1380 s, 1235 m	1070 m	835 s, 780 m	535 m	420 m	355 w
C3	3385 m, 3275 m	1575 s	1365 s, 1240 m	1060 m	840 s, 795 m	520 m	430 m	360 m

**Table 3:** Bond lengths of the ligand.

Bond	Actual	Optimal	Bond	Actual	Optimal	Bond	Actual	Optimal
N(2)–H(11)	1.0117	1.0120	C(8)–H(15)	1.1188	1.1130	N(5)–H(13)	0.9906	1.0120
H(20)–C(10)	1.1242	1.1130	C(9)–C(8)	1.5256	1.5230	H(12)–N(5)	0.9929	1.0120
C(10)–H(19)	1.1246	1.1130	C(7)–N(1)	1.3204	1.2600	C(3)–N(5)	1.3652	1.3690
H(18)–C(9)	1.1167	1.1130	C(7)–C(8)	1.5251	1.4970	C(3)–S(4)	1.6285	1.5760
C(9)–H(17)	1.1169	1.1130	H(14)–N(6)	0.9976	1.0500	N(2)–C(3)	1.4145	1.3690
C(10)–C(9)	1.5399	1.5230	C(10)–N(6)	1.4571	1.4700	N(1)–N(2)	1.3414	
C(8)–H(16)	1.1188	1.1130	C(7)–N(6)	1.4224	1.4620			

**Table 4:** Bond angles of the ligand.

Angle	Actual	Optimal	Angle	Actual	Optimal
H(20)–C(10)–H(19)	108.0742	109.4000	C(7)–C(8)–H(15)	110.1767	109.4100
H(20)–C(10)–C(9)	110.8390	109.4100	C(7)–C(8)–C(9)	105.3224	109.5000
H(20)–C(10)–N(6)	107.3801		N(1)–C(7)–C(8)	128.1179	115.1000
H(19)–C(10)–C(9)	110.4255	109.4100	N(1)–C(7)–N(6)	121.7403	126.0000
H(19)–C(10)–N(6)	111.8302		C(8)–C(7)–N(6)	110.0955	125.3000
C(9)–C(10)–N(6)	108.2648		C(10)–N(6)–H(14)	115.3567	
H(18)–C(9)–H(17)	108.0210	109.4000	C(7)–N(6)–H(14)	116.0931	118.0000
H(18)–C(9)–C(10)	110.5216	109.4100	C(7)–N(6)–C(10)	109.1525	
H(18)–C(9)–C(8)	111.1762	109.4100	H(12)–N(5)–H(13)	119.3842	118.8000
C(10)–C(9)–H(17)	110.4261	109.4100	C(3)–N(5)–H(13)	123.4115	
H(17)–C(9)–C(8)	110.9742	109.4100	H(12)–N(5)–C(3)	117.1111	
C(10)–C(9)–C(8)	105.7415	109.5000	N(5)–C(3)–S(4)	122.0027	124.3000
H(15)–C(8)–H(16)	108.4617	109.4000	N(2)–C(3)–N(5)	120.9560	120.0000
C(9)–C(8)–H(16)	111.3872	109.4100	N(2)–C(3)–S(4)	116.9829	124.3000
C(7)–C(8)–H(16)	110.1951	109.4100	C(3)–N(2)–H(11)	112.0942	117.4000
C(9)–C(8)–H(15)	111.2857	109.4100	N(1)–N(2)–H(11)	120.3309	
C(10)–C(9)–H(17)	110.4261	109.4100	N(1)–N(2)–C(3)	122.2156	

**Table 5:** Atomic charge of the ligand.

Charges	Atoms	Charges	Atoms	Charges	Atoms	Charges	Atoms
N −0.431	[N(1)]	N 0.081	[N(6)]	H 0.068	[H(11)]	H 0.040	[H(16)]
N 0.263	[N(2)]	C 0.222	[C(7)]	H 0.101	[H(12)]	H 0.031	[H(17)]
C 0.233	[C(3)]	C **−**0.122	[C(8)]	H 0.104	[H(13)]	H 0.028	[H(18)]
S −0.696	[S(4)]	C **−**0.066	[C(9)]	H 0.091	[H(14)]	H 0.011	[H(19)]
N −0.049	[N(5)]	C 0.036	[C(10)]	H 0.038	[H(15)]	H 0.018	[H(20)]
